# A novel serum biomarker quintet reveals added prognostic value when combined with standard clinical parameters in prostate cancer patients by predicting biochemical recurrence and adverse pathology

**DOI:** 10.1371/journal.pone.0259093

**Published:** 2021-11-12

**Authors:** Alcibiade Athanasiou, Pierre Tennstedt, Anja Wittig, Ramy Huber, Oliver Straub, Ralph Schiess, Thomas Steuber

**Affiliations:** 1 Proteomedix AG, Zurich-Schlieren, Switzerland; 2 Martini-Klinik, University Hospital Hamburg-Eppendorf, Hamburg, Germany; The University of Texas MD Anderson Cancer Center, UNITED STATES

## Abstract

The objective was to determine the prognostic utility of a new biomarker combination in prostate cancer (PCa) patients undergoing Radical Prostatectomy (RP). Serum samples and clinical data of 557 men who underwent RP for PCa with pathological stage (pT) <3 at Martini Clinic (Hamburg, Germany) were used for analysis. Clinical Grade Group and clinical stage was determined using biopsy samples while tumor marker concentrations were measured in serum using immunoassays. The prognostic utility of the proposed marker combination was assessed using Cox proportional hazard regression and Kaplan-Meier analysis. The performance was compared to the Cancer of the Prostate Risk Assessment (CAPRA) score in the overall cohort and in a low-risk patient subset. A multivariable model comprising fibronectin 1, galectin-3-binding protein, lumican, matrix metalloprotease 9, thrombospondin-1 and PSA together with clinical Grade Group (GG) and clinical stage (cT) was created. The proposed model was a significant predictor of biochemical recurrence (BCR) (HR 1.29 per 5 units score, 95%CI 1.20–1.38, p<0.001). The Kaplan-Meier analysis showed that the proposed model had a better prediction for low-risk disease after RP compared to CAPRA (respectively 5.0% vs. 9.1% chance of BCR). In a pre-defined low risk population subset, the risk of BCR using the proposed model was below 5.2% and thus lower when compared to CAPRA = 0–2 (9%), GG<2 (7%) and NCCN = low-risk (6%) subsets. Additionally, the proposed model could significantly (p<0.001) discriminate patients with adverse pathology (AP) events at RP from those without. In conclusion, the proposed model is superior to CAPRA for the prediction of BCR after RP in the overall cohort as well as a in a pre-defined low risk patient population subset. It is also significantly associated with AP at RP.

## Introduction

Prostate cancer (PCa) is a major cause of cancer-related mortality among men in developed countries [1]. Tumors can be managed through curatives therapies, such as Radical Prostatectomy (RP), which provides excellent cancer control of localized PCa [2]. Approximately 30% of surgically treated men will experience biochemical recurrence (BCR) [3] being at significant risk for clinical cancer progression (metastases) and have the need for institution of systemic therapy.

Clinical stage, pretreatment PSA levels and prostate biopsy Gleason grade have been shown to be reliable and independent predictors of treatment failure [4]. Clinical risk profiles (pre-treatment nomograms (e.g. Kattan [5] or CAPRA score [6]) were designed to identify patients who can safely avoid aggressive therapy or to select potential candidates for neoadjuvant clinical trials.

The usefulness of current models, however, depends on their predictive accuracy. Preoperative PSA levels may reflect primarily benign prostate hyperplasia (BPH) rather than the presence of PCa in populations in which PSA is regularly used for screening. After biopsy, the prediction for aggressiveness of PCa is difficult even with the use of nomograms incorporating clinical information in their algorithm. This is especially for patient with a low and very-low risk of disease progression, for whom active surveillance is becoming a widely adopted strategy. Therefore, there is a compelling need to identify novel markers that are specifically linked to the presence of biologically aggressive PCa for improved prediction of outcome in populations with moderately elevated PSA levels. Because of these current limitations with models based primarily on total PSA levels, we investigated alternative PCa related biomarkers and their association to BCR and adverse pathology (AP) in patients with clinically localized PCa.

In this study we performed univariate and multivariate analysis of multiple protein biomarkers originally discovered in the context of PTEN-mutation using mouse model [7] and proteomics technology. Here we selected 20 biomarker candidates for further evaluation from the originally 39 candidates discovered based on immunoassay reagents availability. The clinical performance of the combination of multiple biomarkers together with the clinical grade group (GG), clinical stage (cT) and PSA were evaluated for the prediction of BCR after RP compared to the CAPRA score. In addition, the association with AP was investigated as well.

## Patients and methods

### Study population

The retrospective cohort included 557 men from the Martini Clinic (Hamburg, Germany). All data were fully anonymized before they were accessed. The study was approved by the local ethics committee and all patients gave written informed consent. All patients were diagnosed with localized PCa, underwent RP and had a clinical stage of cT<3 with or without staging lymphadenectomy. All blood samples were drawn prior RP, eight or more weeks after any prostatic manipulation (DRE, TRUS guided biopsy) and immediately processed and frozen. None of the patients had undergone any additional treatment.

The primary outcome was BCR after RP, defined as any postoperative PSA >0.2 ng/ml [8]. Patients were censored at 5 years of follow-up. The secondary outcome was AP at RP, defined as either a pathological GG3 or greater, pathological stage of pT3a or greater, or positive pathological Node (pN1) [9, 10].

### Assay methods

CE-IVD immunoassays were used for the quantification of CTSD and THBS1 (Proteomedix, Proclarix assays) [11]. Assays were performed according to the manufacturer’s instructions. All other immunoassays were non-IVD immunoassays and composed of either commercially available components from R&D Systems (ATRN, ECM1, LG3BP, LRG1, LUM, MMP9, NCAM1, TIMP1, VEGF, ZAG) or reagents proprietary to Proteomedix (CFH, FN1, HYOU1, ICAM1, OLFM4, POSTN, VTN). Detailed assay reagent sources are listed in the supplementary information (S1 Table). The format used was either ELISA (CTSD, THBS1, CFH, FN1, VTN, POSTN) or Luminex (all other markers). Proprietary recombinant proteins (HYOU1, ICAM1, OLFM4) and commercially available recombinant proteins (all other markers) were used as reference for the calibration of the immunoassays.

In brief, for ELISAs, capture antibodies were coated overnight at 4 °C, washed and blocked for 2 h with BSA-Block solution (Candor GmbH). After washing, the protein (serum sample or standard) diluted in LowCross buffer (Candor GmbH) was added simultaneously with biotinylated antibodies and incubated for 60 min at 37°C with 650 rpm followed by another washing step. Then Streptavidin-HRP conjugate was incubated for 30 min at 37 °C with 650 rpm. After a final washing step, TMB (Enhanced K-Blue Substrate, Neogen) was added for 30 min at 37 °C with 650 rpm and stopped with 1M HCL before being measured at 420 nm.

For Luminex, the assays were performed either measuring proteins in a multiplex format (Mix1: MMP9, NCAM1, ICAM1, LUM, TIMP1; Mix2: ECM1, LG3BP, ZAG; Mix3: ATRN, LRG1) or in a single reaction format (HYOU1, OLFM4). The procedure used was the same as the one described previously [12].

### Statistical methods

The proposed biomarker model for prognosis of patients with BCR was developed as follows: for all 20 markers univariate Cox proportional hazard (CoxpH) on BCR and General Linear Model (GLM) on AP was created. Markers regulated in the same direction (up or down) for BCR and AP were kept for further model building. Step Akaike Information Criteria (StepAIC) selection was then applied using CoxPH on BCR and Glm on AP. Finally, a multivariate CoxPH model was used to create the algorithm of the new proposed model. The goodness-of-fit of the CoxPH model was assessed using the Schoenfeld’s approach [13, 14]. A nonsignificant result for this test indicates no deviation from the proportional hazard assumption, thus the proposed CoxPH model would be robust.

The prognostic utility of the proposed model on BCR was assessed by using the Kaplan-Meier time-to-event approach. Results of the proposed model were compared to NCCN criteria [15] or CAPRA score [16]. For discriminative ability of AP at RP, the two-sided t-test p<0.05 was considered as statistically significant. All statistical analysis was performed using R statistical packages version 4.0.2 and GraphPad PRISM version 6.0.

## Results

### Biopsy outcome

Patient characteristics are displayed in Table 1. Of the 557 men included in the study, the median (min-max) age was of 65 (44–78). The large majority of the patients had a low to intermediate risk of PCa based on NCCN criteria (87% of the population) or CAPRA score (89%). Among the 557 patients, 31% showed an AP event at RP. Fourteen percent of patients had BCR within 5 years. The median follow-up time for those without BCR was 7.0 years (IQR 5.0, 7.4).

**Table 1:** Clinical characteristic of the patients.

General		
All patients, n (%)	557	(100)
Median age at diagnosis, years (range)	65	(44–78)
**Biopsy characteristics**	**n**	**(%)**
≤ 10 ng/ml	460	(83)
10–20 ng/ml	75	(13)
>20 ng/ml	22	(4)
Grade Group		
1	257	(46)
2	169	(30)
3	76	(14)
4	38	(7)
5	17	(3)
Clinical Stage (cT)		
cT1	474	(85)
cT2	83	(15)
NCCN risk		
low	200	(36)
intermediate	282	(51)
high	75	(13)
CAPRA score		
CAPRA 0	2	(0.4)
CAPRA 1	86	(15)
CAPRA 2	143	(26)
CAPRA 3–5	269	(48)
CAPRA 6–10	57	(10)
**Surgical characteristics**	**n**	**(%)**
Grade Group		
1	85	(15)
2	385	(69)
3	76	(14)
4	5	(1)
5	6	(1)
Pathological Stage		
pT2	429	(77)
pT3	128	(23)
Regional Lymph Nodes		
N0	431	(77)
N1	17	(3)
NX	109	(20)
**Progression to aggressive PCa**	**n**	**(%)**
Progression to BCR		
Events	77	(14)
Median years to follow up (range) ^(a)^	7.0	(5.0–7.4)
Progression to AP		
GG>2	84	(15)
pT>2	128	(23)
N>0	17	(3)
Total ^(b)^	170	(31)

^(a)^ Follow up for men who had not experienced an event.

^(b)^ multiple events for the same patient possible.

### Proposed model building

Univariate CoxPH models on BCR and GLMs on AP are shown in Table 2A. Hazard Ratio (HR) and Odd Ratios (OR) comparison ruled out age, ATRN, OLFM4, POSTN and TIMP1 for further model building. Stepwise selection applied for CoxPH on BCR and for GLM on AP, yielded a 10-plex model for BCR (GG, PSA, cT, ECM1, FN1, LG3BP, LUM, MMP9, THBS1 and VTN) and 6-plex model for AP (GG, cT, prostate volume, PSA, LG3BP and LUM). Out of those 11 different variables, the performance of 20 different multivariate CoxPH models combining 6 to 8 variables were tested for discrimination of low versus intermediate and high risk of BCR. Acceptable low risk fraction of BCR was set to be below 5% after 5 years. Finally, the best CoxPH model comprising FN1, LG3BP, LUM, MMP9, THBS1 and PSA together with GG and cT was selected as the new proposed model.

**Table 2:** Uni- and multivariate analysis. (A) Univariate hazard ratio of Cox proportional hazards regression (CoxPH) on Biochemical recurrence after surgery (BCR) and odd ratios of General Linear Model (Glm) on adverse pathology (AP). (B) Multivariate CoxpH on BCR, the proposed model is composed of Grade Group + PSA + cT + LUM + FN1 + LG3BP + MMP9 +THBS1.

**A**		**CoxpH model on BCR**		**Glm model on AP**	
**Marker**	**Units increase**	**HR (95% CI)**	***p*-value**	**OR (95% CI)**	***p*-value**
Age	1 year	0.99 (0.96–1.02)	0.390	1.07 (1.04–1.10)	<0.001
Grade Group	1 unit	1.60 (1.35–1.90)	<0.001	1.67 (1.38–1.99)	<0.001
Prostate volume	10 ml	0.95 (0.84–1.07)	0.380	0.90 (0.81–1.01)	0.064
PSA	1 ng/ml	1.03 (1.01–1.05)	0.010	1.07 (1.03–1.10)	<0.001
Clinical stage (cT)	1	3.06 (1.90–4.93)	<0.001	3.06 (1.85–5.07)	<0.001
ATRN	1 μg/ml	0.99 (0.96–1.02)	0.515	1.01 (0.99–1.04)	0.322
CFH	1 μg/ml	1.00 (1.00–1.01)	0.432	1.00 (1.00–1.01)	0.170
CTSD	100 ng/ml	0.94 (0.79–1.11)	0.469	0.99 (0.86–1.14)	0.869
ECM1	100 ng/ml	0.96 (0.91–1.02)	0.152	0.99 (0.94–1.04)	0.667
FN1	1 μg/ml	1.00 (1.00–1.00)	0.210	1.00 (1.00–1.00)	0.732
LG3BP	1 μg/ml	0.93 (0.83–1.04)	0.195	0.96 (0.89–1.05)	0.377
HYOU1	100 ng/ml	1.75 (0.80–3.83)	0.165	1.05 (0.53–2.10)	0.883
ICAM1	100 ng/ml	1.48 (0.83–2.65)	0.182	1.51 (0.91–2.51)	0.110
LRG1	1 μg/ml	1.09 (0.97–1.22)	0.135	1.12 (0.97–1.30)	0.119
LUM	100 ng/ml	1.04 (0.89–1.23)	0.601	1.09 (0.97–1.23)	0.131
MMP9	100 ng/ml	1.05 (1.02–1.09)	0.002	1.00 (0.97–1.04)	0.972
NCAM1	100 ng/ml	0.91 (0.70–1.18)	0.469	0.99 (0.80–1.23)	0.923
OLFM4	100 ng/ml	1.63 (1.01–2.62)	0.044	0.82 (0.49–1.36)	0.436
POSTN	100 ng/ml	0.79 (0.57–1.11)	0.172	1.02 (0.93–1.11)	0.728
THBS1	1 μg/ml	0.99 (0.97–1.01)	0.319	0.99 (0.98–1.01)	0.579
TIMP1	100 ng/ml	1.09 (0.95–1.26)	0.217	0.99 (0.87–1.15)	0.954
VEGF	1 μg/ml	1.05 (0.27–4.08)	0.949	1.01 (0.32–3.16)	0.990
VTN	1 μg/ml	1.02 (0.99–1.04)	0.170	1.00 (0.98–1.02)	0.966
ZAG	1 μg/ml	1.05 (0.96–1.16)	0.267	1.02 (0.93–1.11)	0.715
**B**		**CoxpH Model for BCR**			
**Model**	**Units increase**	**HR (95% CI)**	***p*-value**	**concordance coefficient**	
CAPRA	1 unit	1.36 (1.21–1.53)	<0.001	0.643	
Grade Group (GG)	1 unit	1.60 (1.35–1.90)	<0.001	0.664	
GG+PSA	5 units	1.25 (1.16–1.35)	<0.001	0.676	
GG+PSA+cT	5 units	1.26 (1.17–1.35)	<0.001	0.698	
Proposed model	5 units	1.29 (1.20–1.38)	<0.001	0.739	

Multivariate analysis of the proposed model for CoxPH on BCR is shown in Table 2B. The proposed model is significantly associated to BCR (HR 1.29 per 5 units score, 95%CI 1.20–1.38, *p*<0.001). Adding PSA to GG, then PSA + cT and finally PSA + cT + five serum markers improved the prediction of BCR by increasing the c-index respectively by 0.012, 0.022 and 0.041. The Schoenfeld’s approach for testing the goodness-of-fit of the CoxPH model showed no difference between the observed covariate and the expected given risk set at that time (S1 Fig). The test was not statistically significant for each of the covariates (*p*>0.07) and for the proposed model (*p* = 0.71, S2 Table). Therefore, we can assume no deviations from the proportional hazard assumptions.

### Kaplan-Meier analysis on BCR prediction

**Fig 1:**
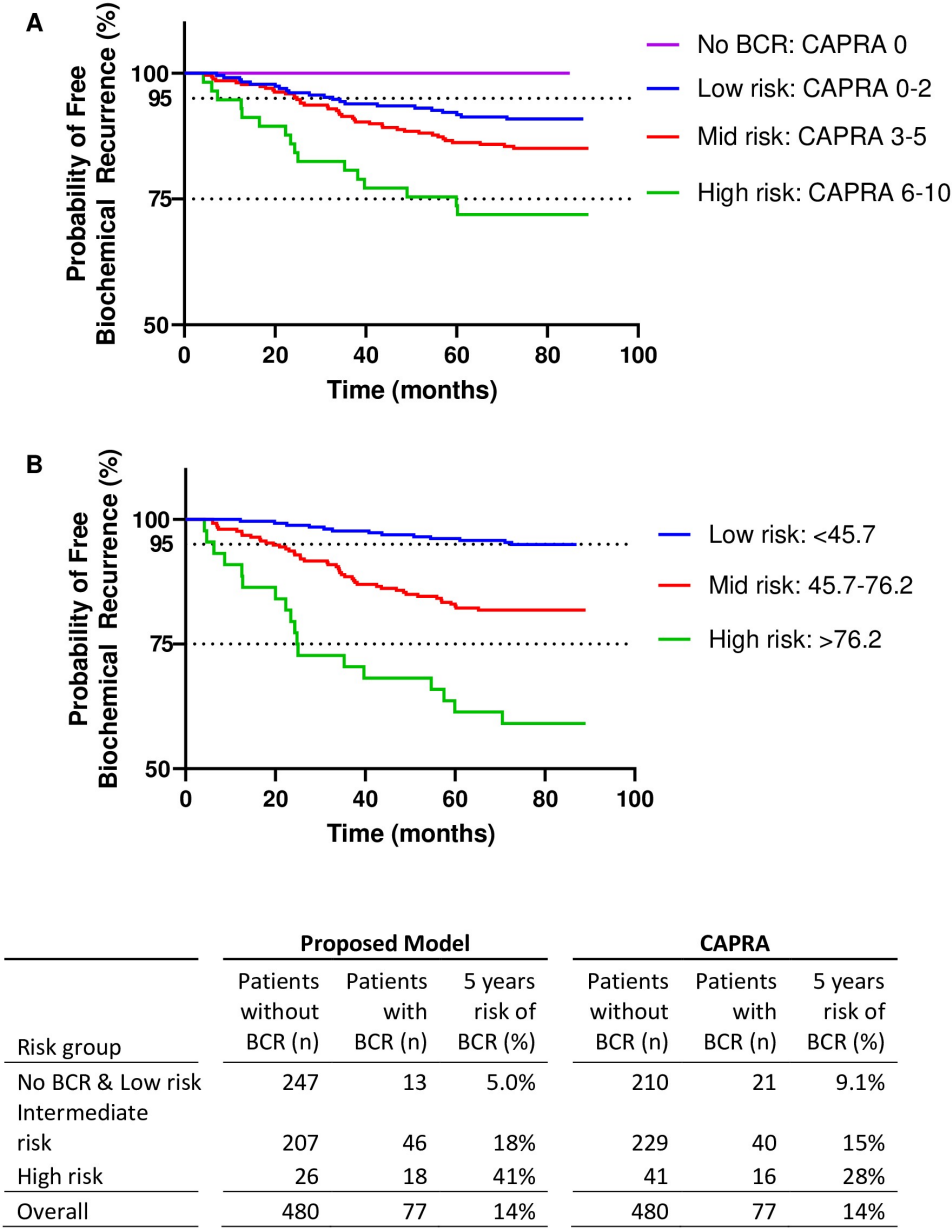
Biochemical recurrence free survival (BCR). (A) CAPRA, (B) proposed model.

The Kaplan-Meier analysis of freedom from BCR is shown in Fig 1. Thresholds for the proposed model were identified in order to stratify the population in low risk (<45.7), intermediate risk (45.7–76.2) and high risk (>76.2) of BCR. For the proposed model, definition of low risk of BCR after 5 year was set to be lower than 5%, and higher than 40% for high risk of BCR.

As a result, the Kaplan-Meier analysis of the overall cohort showed that the proposed model has a better prediction of low-risk BCR after RP compared to CAPRA (respectively 5.0% vs. 9.1% chance of BCR, for n = 247 and n = 210 patients). Those results show the superior ability of the proposed model to discriminate patients with the low risk of BCR. These findings were similar when applying the proposed model in cohorts with pre-defined low risk of BCR by selecting patients with CAPRA<2 (n = 231), GG<2 (n = 257) or NCCN = low risk (n = 200). Results are shown in Table 3. Here, the risk of BCR using the low-risk cutoff of the proposed model (<45.7) was below 5.2% (n >142 patients) in all three subgroups and thus lower when compared to CAPRA = 0–2 (9%), GG<2 (7%) and NCCN = low-risk (6%) subsets.

**Table 3:** Performance of the proposed model for biochemical free survival (BCR) in CAPRA 0–2, NCCN low and Grade Group 1 patient population.

Risk of BCR	Threshold from Proposed Model	CAPRA 0–2 Patients ^(a)^ (n, %BCR risk)	NCCN Low Patients (n, %BCR risk)	Grade Group 1 Patients (n, %BCR risk)
Low Risk	<45.7	n = 165, 4.8% BCR	n = 142, 4.9% BCR	n = 192, 5.2% BCR
Mid Risk	45.7–76.2	n = 62, 16% BCR	n = 58, 7% BCR	n = 63, 9.5% BCR
High Risk	>76.2	n = 4, 50% BCR	none	n = 2, 50% BCR
Overall	n/a	n = 231, 9% BCR	n = 200, 5.5% BCR	n = 257, 7% BCR

^(a)^ n = 2 patients with CAPRA = 0.

### Discrimination of adverse pathology

**Fig 2:**
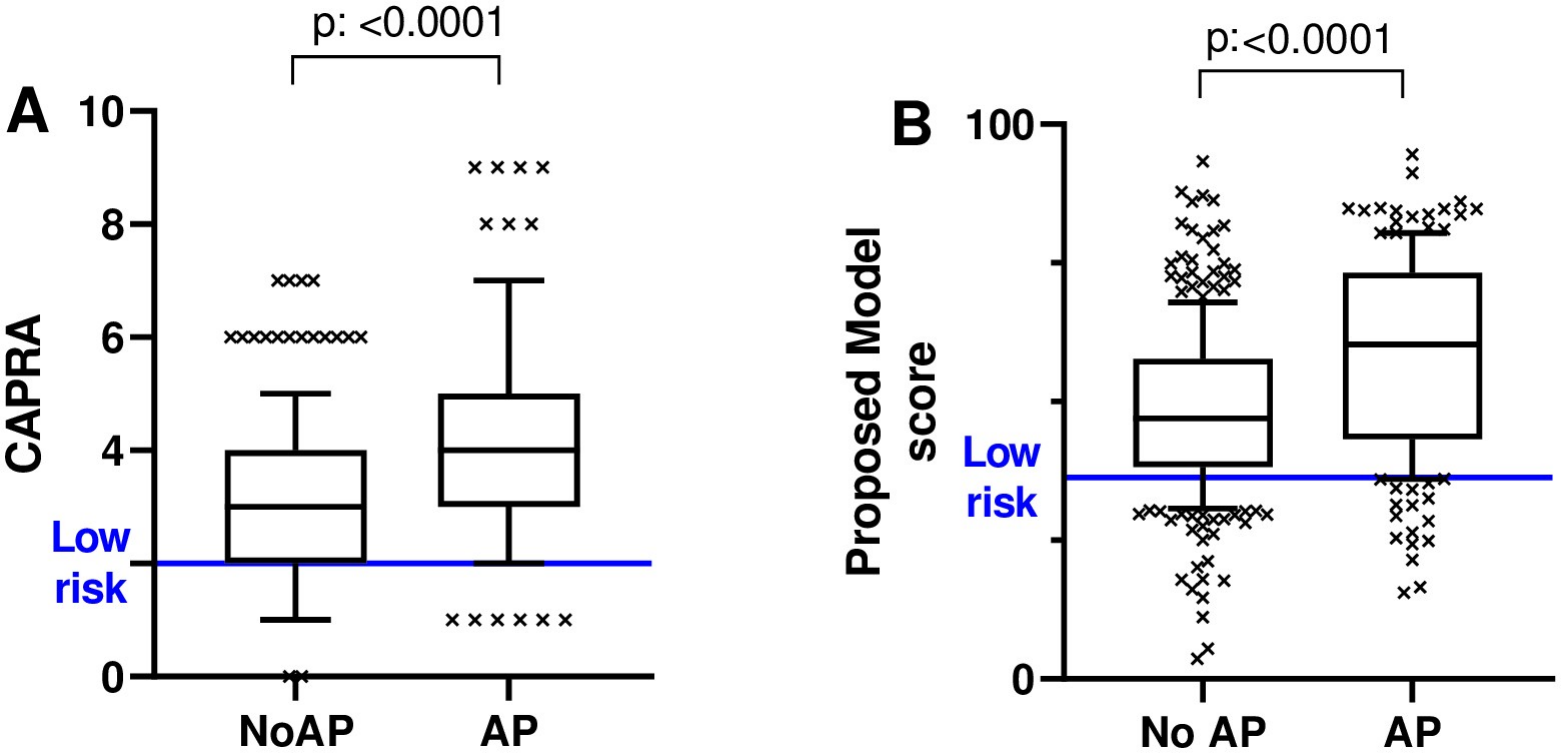
Association with adverse pathology (AP) features. (A) CAPRA and (B) proposed model.

When applying a threshold <36, the proposed model is significantly associated with AP at RP (*p*<0.001; Fig 2) as well as with the three single AP events (*p*<0.001 for GG>2, pT>2 and pN1; S2 Fig). The clinical performance for the prediction AP was superior to CAPRA (S3 Table): when applying a threshold CAPRA<2 and a cutoff of <36 for the proposed model, the specificity between the two models turned out to be significantly better for the proposed model (*p* = 0.04), while the difference in sensitivity was just no significant (*p* = 0.06).

## Discussion

The ability to assess prognosis of PCa is critical for the management of men undergoing a RP. The difficulty of the prediction of PCa is enhanced by the variety of adverse outcome linked to PCa progression: BCR, AP, metastasis or death. The ideal prognostic model would need to cover all these aspects in order to help on the decision making for possible post-operative treatments. The current stratification of the risk in clinical practice remains fairly poor. Various free nomograms (i.e. CAPRA, d’Amico score) have been developed based on pathological outcome. Commercially available tests like the CCP-score, a tissue based genomic test of 31 call cycle progression genes or the GPS-score, a test based on the RNA expression of 17 genes, could also stratify the risk of PCa progression, as it was shown in multiple studies for CAPRA [16], CCP [17] or GPS [18]. However, the difficulty to identify one logical threshold, with which to guide treatment across different cohorts remains challenging.

In this study we evaluated the prognostic usability of a new proposed model for the assessment of BCR after RP, and AP at RP. The performance of the proposed model was compared to the CAPRA. All patients from the study population (n = 557) had a clinical stage below 3.

As expected, the prognostic capability of CAPRA for BCR was limited in the cohort. The too high among of the low-risk patient population (CAPRA 0–2, 9.1% BCR, n = 210) and too low amount of patient without BCR (CAPRA = 0, n = 2) makes it difficult to be used from clinicians for safe treatment guidance of the patients.

Here we first developed a model with protein biomarkers originally discovered in the context of PTEN-mutation using mouse model [7]. The selection of markers was performed in two steps; first a univariate analysis ruled out markers that were not regulated the same way in AP and BCR as this has a negative impact on the reproducibility of the algorithm. All remaining variables, including all non-significant ones, were kept for a further StepAIC selection using a coxPH model, as such markers can contribute to a multivariate model [19]. AIC uses the number of variable as well as the model’s maximum log-Likelihood as fitting criteria and coxPH model is a regression model where coefficients are calculated for every protein biomarker. Thus, the model does not use a threshold approach for selection the protein biomarker. Finally, the final selected multivariable model is combining THBS1, LUM, FN1, MMP9, GL3BP together with PSA, clinical GG and clinical stage. Here also, all eight variables were kept into the model, including the nonsignificant ones with a rather small impact on the HR or c-index, as these variables coefficients do have an impact on the other variables coefficients of the model. The elimination of a nonsignificant variable can lead to the adjustment of another significant variable that might change from “significant” to “nonsignificant”, and hence leading to the elimination of that significant variable in a later step [20].

The proposed model could significantly (p<0.001) discriminate patients with Adverse Pathology (AP) events at RP and was a significant predictor of BCR (HR 1.29 per 5 units score, 95%CI 1.20–1.38, p<0.001). Those findings are supported with the analysis of the c-index, which increases when adding the five biomarkers to the PSA GG and cT. The association of the new serum markers with PCa was already described in the literature (THBS1 [12], MMP9 [21], LUM [22] FN1 [23] GL3BP [24]).

The proposed model shows a superior prediction of BCR after RP compared to CAPRA. It could predict no risk of BCR for 14.4% of the population, where CAPRA predicted less than 0.1% with CAPRA = 0. It could also predict 5.0% recurrence if applying a low-risk threshold of below 45.7 (n = 247) compared to 9.1% for low-risk CAPRA = 0–2 (n = 210). A risk of less than 5% could be considered as fairly low, putting patients at an appreciable risk of BCR after RP.

Among the different low-risk patient population defined as CAPRA = 0–2, NCCN = low and GG<2, the proposed model was with less than 5.2% risk of BCR again slightly superior to CAPRA (9% risk of BCR), NCCN (6%) and GG (7%).

Only 14% patients had a biochemical progression (BCR) within 5 years. This due to the selection criteria excluding patients undergoing neo- and adjuvant treatment as well as selecting pT<3 patients. Nevertheless, the cohort used for this study can be considered as representative of a low-risk patient population, where risk stratification remains especially challenging. The cohort is comparable to the ones used in other studies, also assessing various models on BCR risk after RP [25].

The present study has some limitations that should be noted. The main limitation is that the proposed model was trained on a single retrospective cohort, restricted to one single centre, with mainly Caucasian men. A generalization of the model to more diverse populations is therefore limited. Additionally, another limitation is the lack of proper validation of the model. Even if the goodness-of-fit of the CoxPH model was assessed using the Schoenfeld’s approach, performance of the proposed model and its selected threshold cannot be extrapolated when applied to another independent cohort. Finally, we could show that the proposed model was significantly associated only with BCR and AP. The association to other relevant prognostic endpoints (i.e death or metastasis) could not be assessed within this cohort.

In conclusion the proposed model improved the clinical stratification of BCR-risk and AP of men undergoing prostatectomy. The model could potentially better guide treatment selection, but validation studies should be performed in independent cohorts in order to validate the model.

## Supporting information

S1 Fig Schoenfeld residual analysis. No deviations from the proportional hazard assumptions can be assumed if the residuals are flat and centered about zero.
(TIF)

S2 Fig Adverse pathology. Association of the proposed model to different Adverse Pathology events.
(TIF)

S1 Table List of components used for the immunoassays. (TIF)

S2 Table Schoenfeld’s residual analysis of the CoxPH model. Correlation between covariate and the expected given risk set at that time.
(TIF)

S3 Table Clinical performance for the prediction of Adverse pathology. (TIF)
