# Supplementary figures and images for: A novel serum biomarker quintet reveals added prognostic value when combined with standard clinical parameters in prostate cancer patients by predicting biochemical recurrence and adverse pathology
Source: PLoS One. 2021 Nov 12;16(11):e0259093. doi: 10.1371/journal.pone.0259093 (PMC8589165; doi:10.1371/journal.pone.0259093)

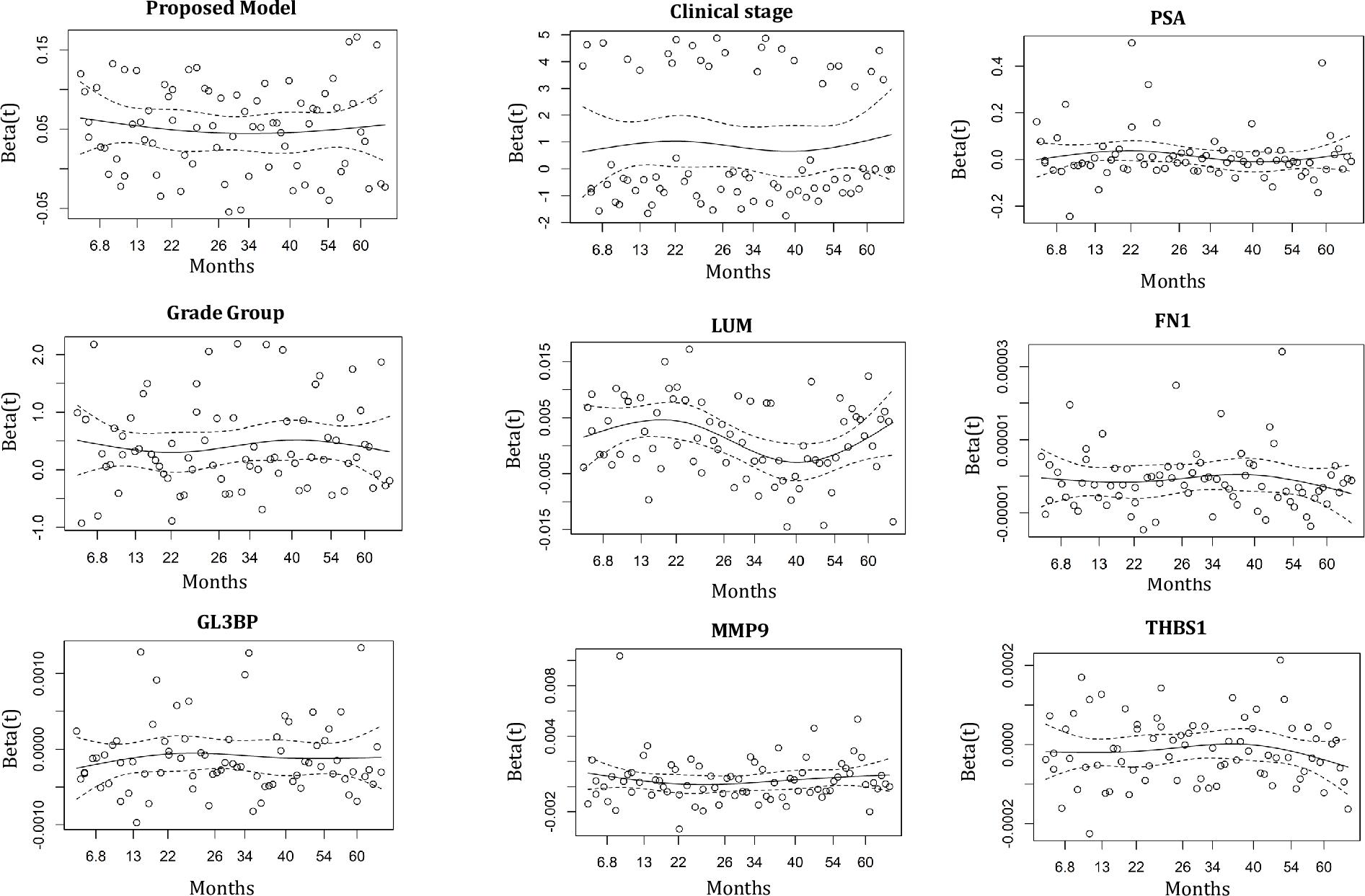

Supplement: S1 Fig — No deviations from the proportional hazard assumptions can be assumed if the residuals are flat and centered about zero. (TIF) [file pone.0259093.s001.tif]

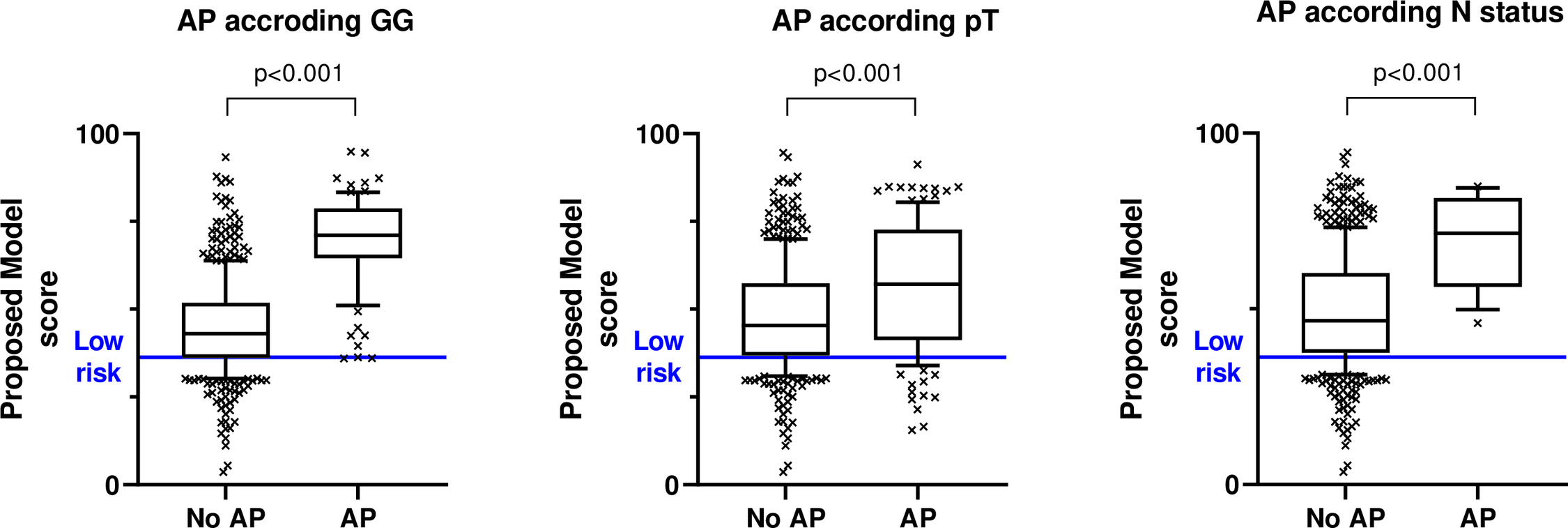

Supplement: S2 Fig — Association of the proposed model to different Adverse Pathology events. (TIF) [file pone.0259093.s002.tif]

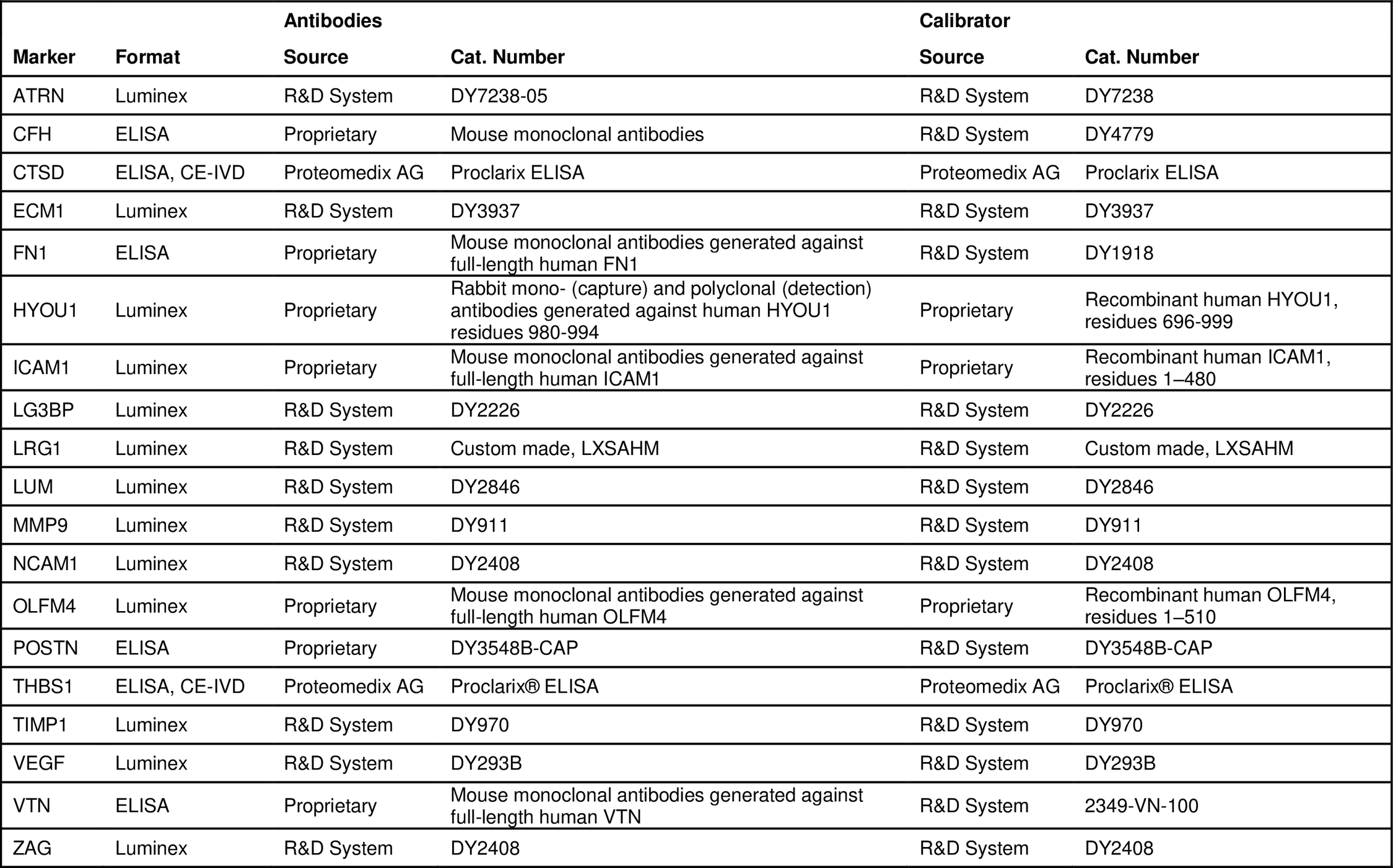

Supplement: S1 Table — (TIF) [file pone.0259093.s003.tif]

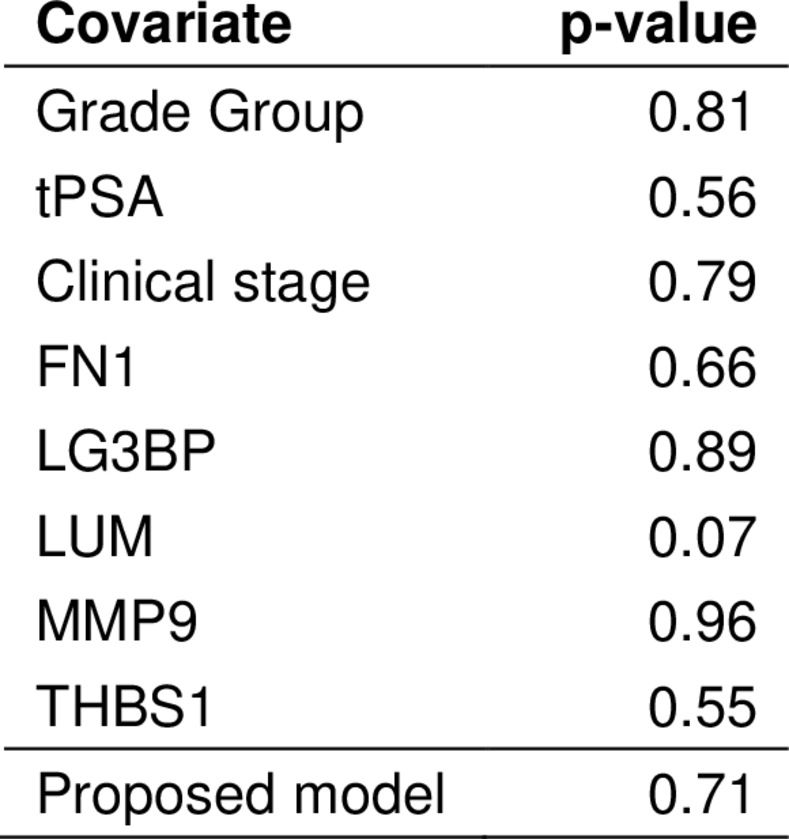

Supplement: S2 Table — Correlation between covariate and the expected given risk set at that time. (TIF) [file pone.0259093.s004.tif]

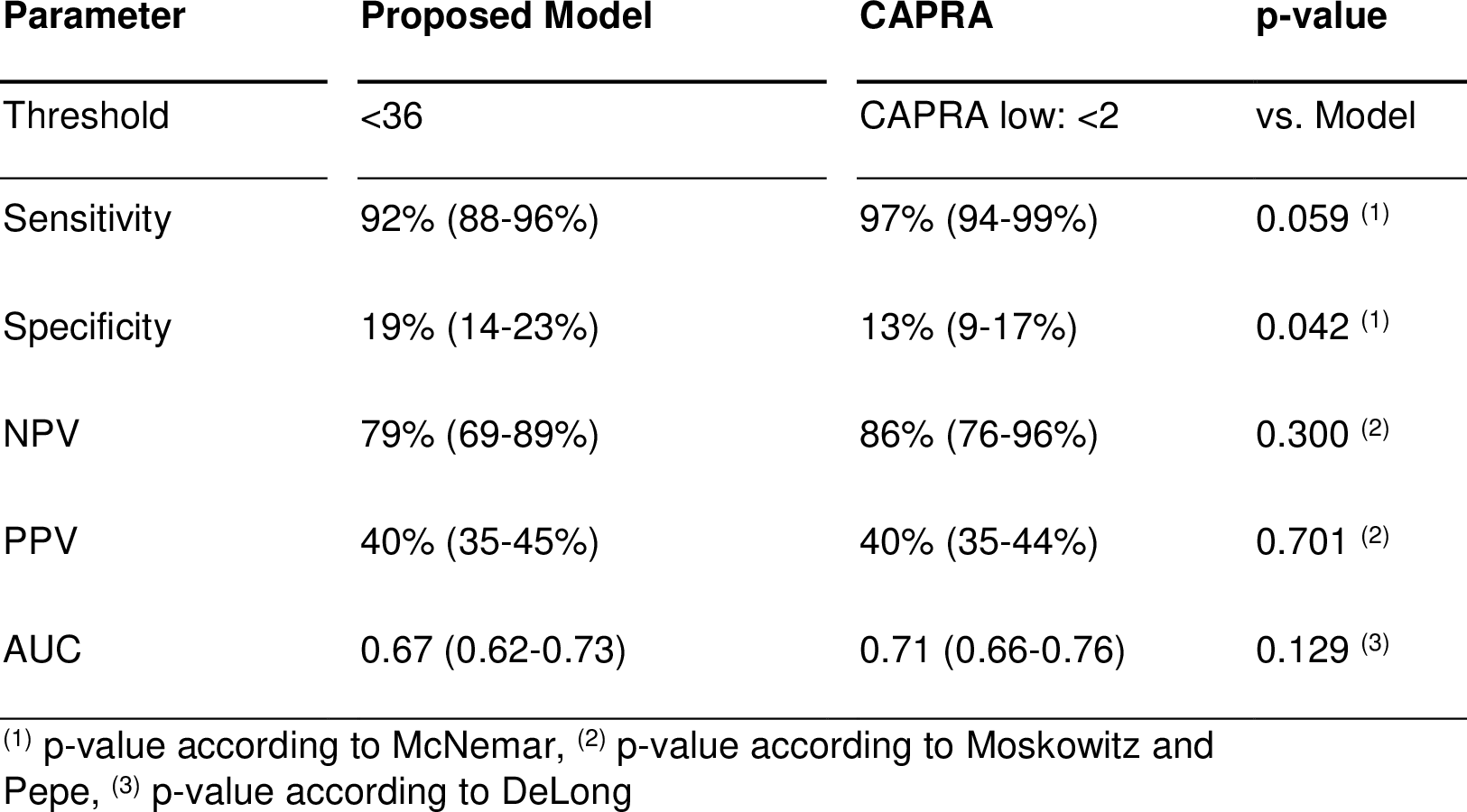

Supplement: S3 Table — (TIF) [file pone.0259093.s005.tif]
